# Significance of non-standard Philadelphia chromosomes in chronic granulocytic leukaemia.

**DOI:** 10.1038/bjc.1981.146

**Published:** 1981-07

**Authors:** A. M. Potter, A. E. Watmore, P. Cooke, J. S. Lilleyman, R. J. Sokol

## Abstract

One hundred and nineteen unselected and similarly treated patients with Ph1-positive chronic granulocytic leukaemia (CGL) had the precise nature of their chromosome rearrangements producing the Ph1 studied to determine whether this had any clinical relevance. Eighteen (15%) did not have the usual 9/22 translocation and these, by life-table analysis, had a significantly shorter benign phase of their disease than the others (P less than 0.01). It further appeared that possession of a non-standard Ph1 was related to age, in that whereas only 24 patients were over 60 at diagnosis, 9 (33%) had a non-9/22 translocation (P less than 0.01). As the duration of the benign phase seemed to be shorter in those over 60 irrespective of Ph1 type (P less than 0.01), the questions arose whether non-standard PhI chromosomes were simply occurring in older patients or whether they were affecting prognosis independently. Their independent effect was suggested by the 11 patients under 60 with a non-9/22 Ph1 who still had a significantly shorter benign phase than the 84 of similar age with a standard Ph1 (P less than 0.01). It is concluded that the myeloid karyotype can provide prognostic as well as diagnostic information in patients with CGL.


*Br. J. Cancer* (1981) **44**, 51

### SIGNIFICANCE OF NON-STANDARD PHILADELPHIA CHROMOSOMES IN CHRONIC GRANULOCYTIC LEUKAEMIA

A. M. POTTER*, A. E. WATMORE*, P. COOKE†, J. S. LILLEYMAN‡ AND R. J. SOKOL§

*From the Departments of *Human Genetics and ‡Haematology, Sheffield Children's Hospital, †Department of Cytogenetics, City Hospital, Nottingham and §The Trent Regional Blood Transfusion Service*

Received 4 February 1981 Accepted 27 March 1981

**Summary.—One hundred and nineteen unselected and similarly treated patients with Ph¹-positive chronic granulocytic leukaemia (CGL) had the precise nature of their chromosome rearrangements producing the Ph¹ studied to determine whether this had any clinical relevance. Eighteen (15%) did not have the usual 9/22 translocation and these, by life-table analysis, had a significantly shorter benign phase of their disease than the others ($P<0{\cdot}01$). It further appeared that possession of a non-standard Ph¹ was related to age, in that whereas only 24 patients were over 60 at diagnosis, 9 (33%) had a non-9/22 translocation ($P<0{\cdot}01$).**

**As the duration of the benign phase seemed to be shorter in those over 60 irrespective of Ph¹ type ($P<0{\cdot}01$), the question arose whether non-standard PhI chromosomes were simply occurring in older patients or whether they were affecting prognosis independently. Their independent effect was suggested by the 11 patients *under* 60 with a non-9/22 Ph¹ who still had a significantly shorter benign phase than the 84 of similar age with a standard Ph¹ ($P<0{\cdot}01$). It is concluded that the myeloid karyotype can provide prognostic as well as diagnostic information in patients with CGL.**

CHRONIC GRANULOCYTIC LEUKAEMIA (CGL) is associated with possession of the Philadelphia chromosome (Ph¹, a characteristically altered Number 22) and is clinically typified as a biphasic disease with a benign phase of about 3 years followed by malignant transformation and subsequent survival of about 3 months.

The commonest chromosome rearrangement to produce Ph¹ is a 9/22 translocation (Rowley, 1973) but subsequent authors have shown that in a minority of patients other rearrangements can produce similar appearances in Chromosome 22 (Hayata *et al.*, 1973; Gahrton *et al.*, 1974). Although such non-standard Ph¹s are not thought relevant to the clinical course of the disease by Sandberg (1980) we report here a large series which appears to contradict this belief.

### PATIENTS AND METHODS

The patients were collected over 5 years, and all referred cases of Ph¹-positive CGL which were successfully banded were included in the series. So-called Ph¹-negative CGL, and those appearing as Ph¹-positive acute leukaemia were excluded. Ages ranged from 14 to 76 years with a mean of 46·5. There were 59 males and 60 females.

Of the 119 patients, 103 were first karyotyped during the clinical benign phase and 16 were not investigated until after malignant transformation.

Marrow samples were studied when possible, otherwise unstimulated blood samples

Correspondence: A. M. Potter, Centre for Human Genetics, Langhill, 117 Manchester Road, Sheffield S10 5DN.

were used, and spleens were examined from the cases with splenectomy. Marrow samples were incubated in TC 199 for 2 h with 0·1 μg/ml colcemid, swollen with 0·075M KCl and fixed with 3:1 ethanol/glacial acetic acid. Slides were air-dried, stained with Leishman stain and banded (Seabright, 1971).

Cell suspensions from blood samples or spleens were incubated in 10 ml of culture medium (3 parts foetal calf serum: 7 parts TC 199) for 64 h, and for a further 6 h with 0·1 μg/ml colcemid. Harvesting and slide preparation were the same as with marrow samples.

All patients were treated during the benign phase with continuous low-dose busulphan, interrupted as considered necessary by the attending physician. Those few patients who could not tolerate this drug were treated similarly with myelobromol or hydroxyurea. Treatment during the malignant phase varied considerably from patient to patient and centre to centre.

The date of malignant transformation for a given patient was determined in all cases by the attending physician. This was decided on clinical grounds, which included loss of disease control and, in some cases, the appearance of large numbers of blast cells. For the purpose of this study, cytogenetic criteria of transformation were not used. Minimum time of follow-up was 16 months. The duration of the benign phase for the various groups was compared by the life-table and logrank methods of Peto *et al.* (1977). Patients who died in benign phase CGL have not been used for the statistical analysis. This includes Cases 67 and 105 with non-standard Ph[1] translocations.

### RESULTS

Ninety-nine out of 119 cases (83%) had a standard 9/22 Ph[1] translocation. Details of 18 non-standard Ph[1] translocations (15%) are given in the Table, which shows an apparently random involvement of chromosomes. Three of these cases (23, 47 and 69) have been included in a previous publication (Potter *et al.*, 1975). Two Ph[1] cases (2%) showing deletion of Chromosome 22 were also found, and included with the non-standard Ph[1] translocations in this study. Mean age at diagnosis of the non-standard Ph[1] cases was 54·3 years, compared with 44·9 years for cases with a 9/22 translocation.

By life-table analysis the duration of the benign phase was significantly shorter for the non-standard Ph[1] patients (median 20 months) than for those with a standard Ph[1] (median 43 months) ($P < 0{\cdot}01$). It also appeared that non-standard Ph[1] translocations were associated with older patients as, of 24 patients over 60 at diagnosis, 9 (37·5%) had a non-standard

TABLE.—*Philadelphia chromosomes not simply 9/22 translocations*

| Case No. | Age (yrs) | Ph[1] translocation | Duration of benign phase (months) |
|---|---|---|---|
| 4 | 52 | t(3;9;22)(p21;q34;q11) | 20 |
| 10 | 73 | t(C;9;22)(q34;q11) | > 16 Alive and well |
| 23 | 47 | t(6;9;22)(p21;q34;q11) | 18 |
| 40 | 42 | t(5;9;22)(q13;q34;q11) | > 48 Alive and well |
| 47 | 69 | t(6;9;22)(p21;q34;q11) | 15 |
| 67 | 68 | t(3;4;9;11;22)(p21;q34;q13;q11) | Died in benign phase |
| 69 | 65 | t(9;13;15;22)(q34;q14;q22;q11) | 45 |
| 82 | 57 | t(9;15;22)(q34;q15;q11) | 17 |
| 90 | 68 | t(9;13;22)(q34;q22;q11) | 18 |
| 124 | 63 | t(9;15;22)(q34;q15;q11) | > 20 Alive and well |
| 13 | 60 | t(6;22)(p25;q11) | > 19 Alive and well |
| 21 | 57 | t(12;22)(q24;q11) | 14 |
| 30 | 49 | t(12;22)(p13;q11) | 26 |
| 32 | 19 | t(17;22)(q25;q11) | 12 |
| 33 | 35 | t(3;22)(p21;q11) | 10 |
| 59 | 35 | t(7;22)(p22;q11) | 48 |
| 63 | 62 | t(15;22)(p11;q11) | 23 |
| 66 | 52 | t(11;22)(p15;q11) | 31 |
| | | Deletions | |
| 6 | 52 | del (22) (q11) | > 16 Alive and well |
| 105 | 61 | del (22) (q11) | Died in benign phase |

FIGURE.—Life table of duration of benign phase of CGL for 3 groups of patients: (A) Patients < 60 yrs with 9/22 Ph[1] (N = 84); (B) Patients > 60 yrs with 9/22 Ph[1] (N = 15); (C) Patients < 60 yrs with non-standard Ph[1] (N = 11). B and C are both significantly different from A ($P < 0{\cdot}01$, logrank). % Untransformed CGLs refers to the proportion of patients continuing in the clinical benign phase of their disease. Open circles represent patients still in that benign phase.

Ph[1] compared to only 11 (11·6%) of those < 60 ($P < 0{\cdot}01$, $\chi^2$). As those aged 60 and over at diagnosis had a significantly shorter benign phase than the rest, irrespective of chromosome status (median again 20 months *vs* 43 months, $P < 0{\cdot}01$), the question arose whether a non-standard Ph[1] simply occurred more frequently in older patients who had a worse prognosis or whether it had any independent prognostic value.

An attempt to answer this was made by comparing the duration of the benign phase in 4 subgroups of patients; (A) 84 under 60 at diagnosis with a typical Ph[1], (B) 15 × 60 + at diagnosis with a typical Ph[1], (C) 11 under 60 at diagnosis with a non-standard Ph[1], and (D) 7 × 60 + at diagnosis with a non-standard Ph[1]. From this it was seen that patients in Groups B, C and D all had a significantly shorter benign phase than patients in Group A, but did not appear to differ from each other. The difference between Group A and Groups B and C is shown graphically in the figure. Group D had a median duration of benign phase of 19 months, and did not appear different in any way from Group B; in other words all those 60 and over had a poor prognosis irrespective of the Ph[1] translocation. The presence of non-standard Ph[1] under 60, however, appeared to be associated with a more rapidly evolving disease—a prognostic effect presumably masked by age.

### DISCUSSION

The nature and significance of unusual Ph[1] translocations have recently been the subject of a multicentre review (Sandberg, 1980) which concluded that the survival of patients with such translocations did not differ significantly from that of patients with the standard 9/22 translocation. Our data seem to contradict this for, although we used the duration of the benign phase rather than survival as our prognostic yardstick, the clear impression is given that non-standard Ph[1] chromosomes tend to be associated with a worse prognosis. They also seem associated with older patients who, in this series, had a worse prognosis irrespective of karyotype, so age may have a stronger influence on outlook.

Our methods might seem open to criticism, in that it could be argued that the duration of the benign phase is hard to measure and depends on the bias of the managing physician deciding when transformation has occurred. This we acknowledge, but would emphasize that such bias would affect all groups of patients equally (and thus be self-cancelling) and that while it is hard to define in words, the event of transformation is frequently obvious in clinical practice. The benign phase duration seems totally unaffected by variations in current conservative treatment.

It is possible that, within the cytogenetic heterogeneity of the non-standard Ph[1]s we have described, there exists a better defined subgroup which is perhaps responsible for the observed effect on prognosis of the group as a whole. Numbers are too small to be other than suggestive, but it may be that the complex translocations arise more frequently in older patients than do simple translocations involving a chromosome other than a 9. Our data would support this, but much greater numbers will be needed to answer the question with confidence. Meanwhile, any patient with a non-standard Ph[1] should be regarded as potentially having a worse outlook than those with an uncomplicated 9/22 translocation.

The authors wish to acknowledge the Physicians of the Trent Region for their assistance and the referral of patients for this series.

A.E.W. is in receipt of a grant from the Special Trustees of the Former United Sheffield Hospitals and J.S.L. is the holder of a grant from the Leukaemia Research Fund.

### REFERENCES

Gahrton, G., Zech, L. & Lindsden, J. (1974) A new variant translocation (19q+;22q−) in chronic myelocytic leukaemia. *Exp. Cell Res.*, **86**, 214.

Hayata, I., Kakati, S. & Sandberg, A. A. (1973) A new translocation related to the Philadelphia chromosome. *Lancet*, ii, 1385.

Peto, R., Pike, M. C., Armitage, P. & 7 others (1977) Design and analysis of randomized clinical trials requiring prolonged observation of each patient. II. Analysis and examples. *Br. J. Cancer*, **35**, 1.

Potter, A. M., Sharp, J. C., Brown, M. J. & Sokol, R. J. (1975) Structural rearrangements associated with the Ph[1] chromosome in chronic granulocytic leukaemia. *Humangenetik*, **29**, 223.

Rowley, J. D. (1973) A new consistent chromosomal abnormality in chronic myelogenous leukaemia identified by quinacrine fluorescence and Giemsa staining. *Nature*, **243**, 290.

Sandberg, A. A. (1980) Chromosomes and causation of human cancer and leukaemia: XL. The Ph[1] and other translocations in CML. *Cancer*, **46**, 2221.

Seabright, M. (1971) A rapid banding technique for human chromosomes. *Lancet*, ii, 971.


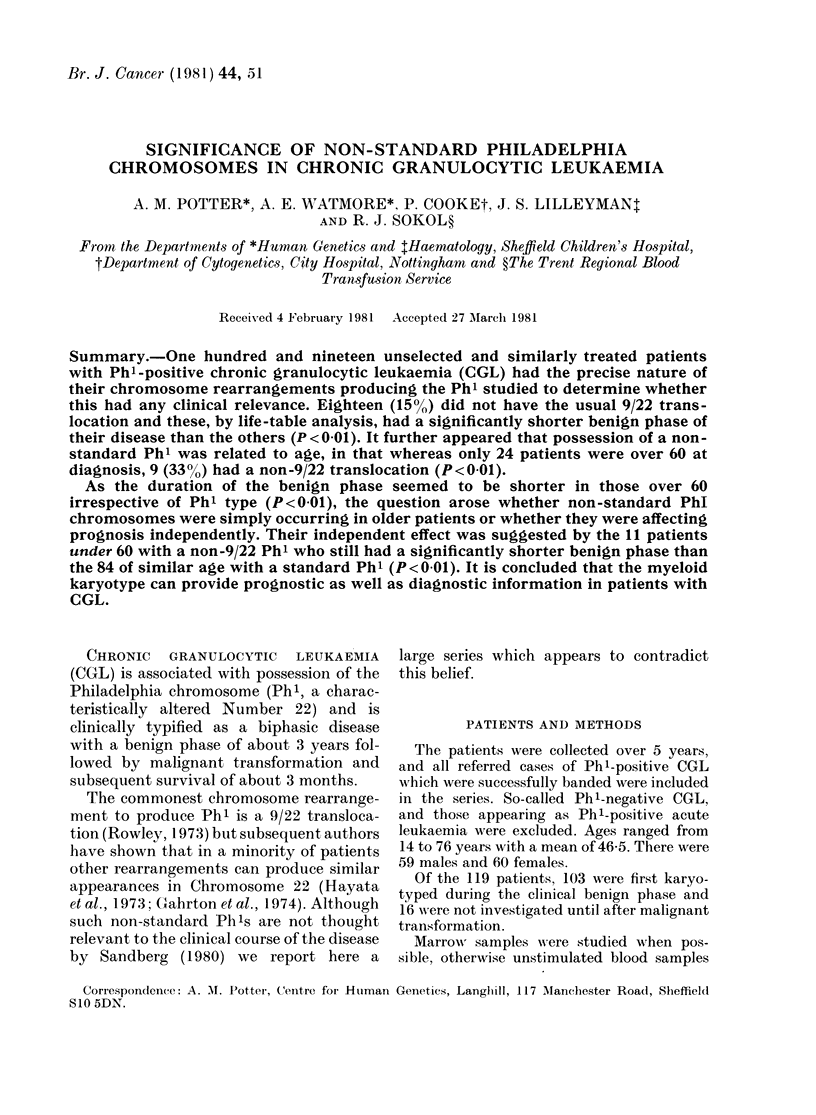

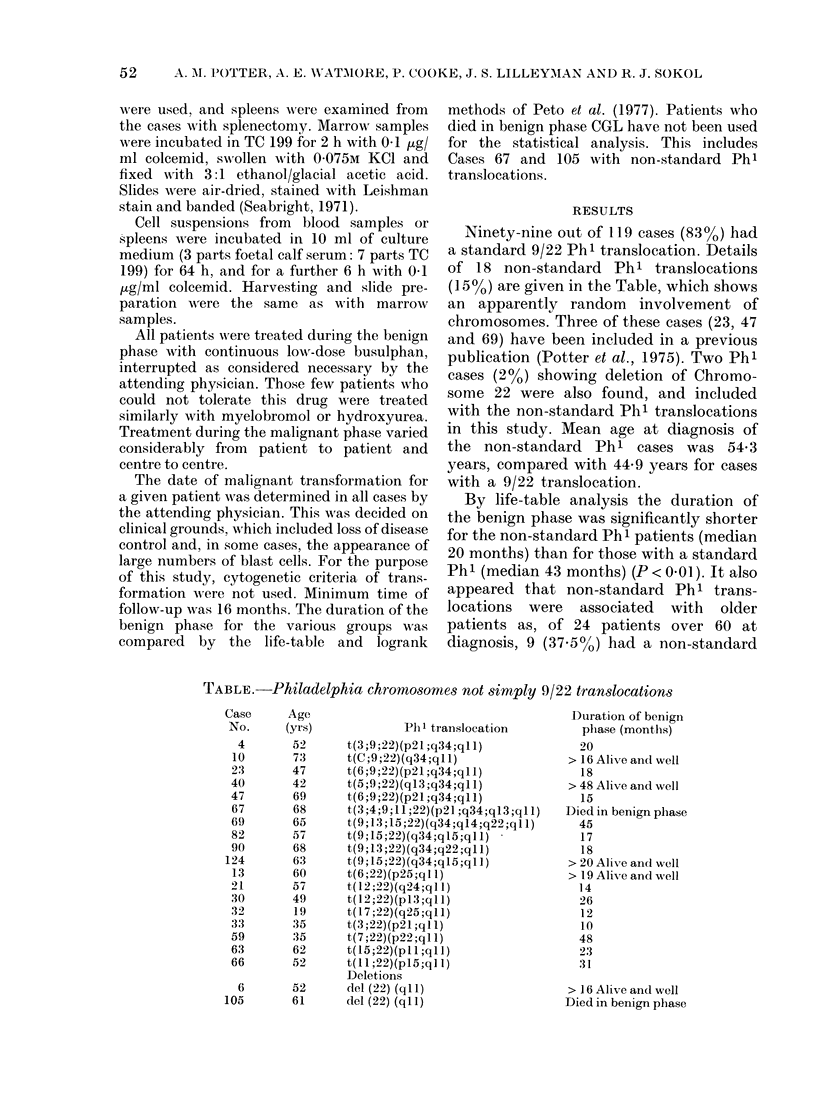

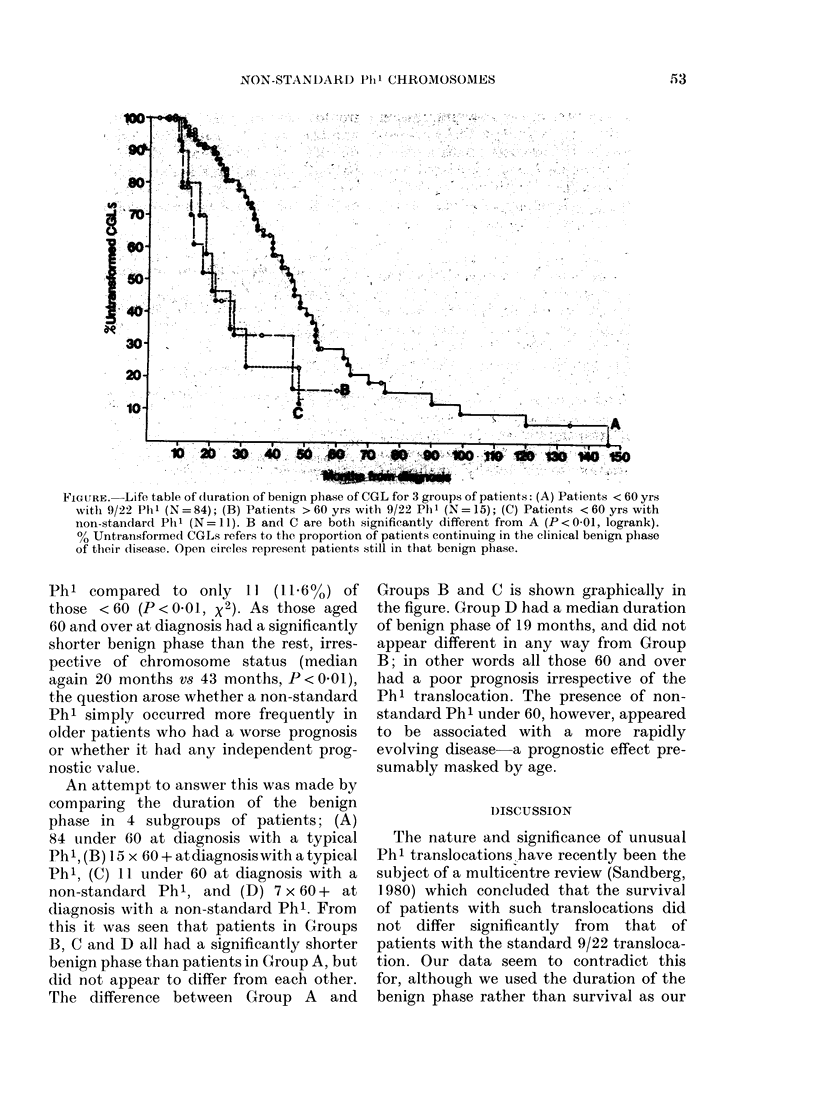

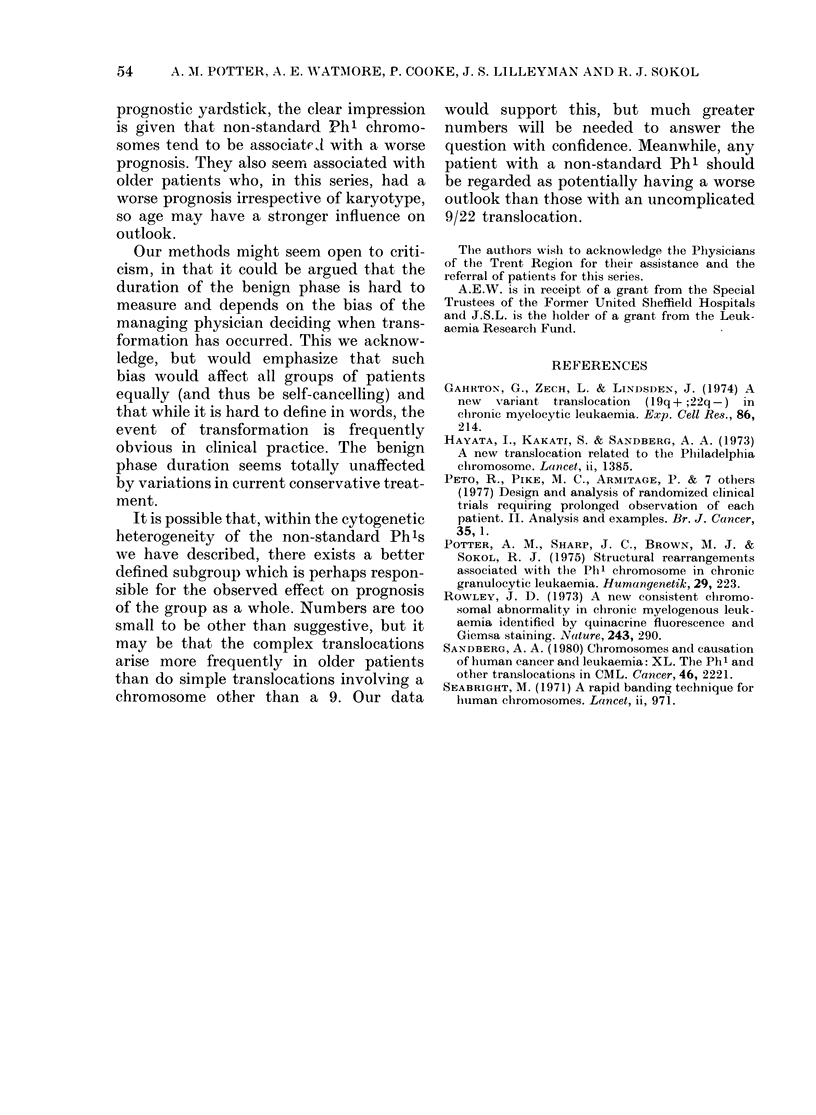


## References

[OCR_00399] Gahrton G., Zech L., Lindsten J. (1974). A new variant translocation (19q plus, 22q minus) in chronic myelocytic leukemia.. Exp Cell Res.

[OCR_00405] Hayata I., Kakati S., Sandberg A. A. (1973). Letter: A new translocation related to the Philadelphia chromosome.. Lancet.

[OCR_00417] Potter A. M., Sharp J. C., Brown M. J., Sokol R. J. (1975). Structural rearrangements associated with the Ph1 chromosome in chronic granulocytic leukaemia.. Humangenetik.

[OCR_00423] Rowley J. D. (1973). Letter: A new consistent chromosomal abnormality in chronic myelogenous leukaemia identified by quinacrine fluorescence and Giemsa staining.. Nature.

[OCR_00429] Sandberg A. A. (1980). Chromosomes and causation of human cancer and leukemia: XL. The Ph1 and other translocations in CML.. Cancer.

[OCR_00434] Seabright M. (1971). A rapid banding technique for human chromosomes.. Lancet.

